# Stem cell derived neural organoid approaches for neurological diseases

**DOI:** 10.4103/NRR.NRR-D-25-01004

**Published:** 2025-12-30

**Authors:** Rosalie Elvira, Xiao-Xue Dong, Alfred Sun Xuyang, Wai Hon Chooi, Huck Hui Ng, Hongyan Wang, Yun-Cheng Wu, Eng King Tan, Zhi Dong Zhou

**Affiliations:** 1 National Neuroscience Institute of Singapore, Singapore; 2 Department of Neurology, Shanghai General Hospital, Shanghai Jiao Tong University School of Medicine, Shanghai, China; 3 Signature Research Program in Neuroscience and Behavioral Disorders, Duke-NUS Graduate Medical School Singapore, Singapore; 4 Genome Institute of Singapore, Singapore; 5 Department of Neurology, Singapore General Hospital, Singapore

**Keywords:** neural differentiation, neural organoids, neurogenesis, neurological diseases, stem cells, three-dimensional models

## Abstract

Traditional two-dimensional cultures and animal models often fall short in capturing the complexities of neurodevelopmental and neurodegenerative diseases. However, recently developed neural organoid approaches, three-dimensional structures derived from human pluripotent stem cells, have become powerful tools for modeling human neuronal development and disease. Unlike traditional models, neural organoids provide significant insights and improved modeling capabilities. Here, we explore various types of neural organoids in disease modeling and outline distinct protocols for generating each type, including specific patterning methods, growth factors, and differentiation durations. The potential and advantages of co-culturing neural organoids with other cells and tissues are also discussed. While neural organoids have already made significant contributions to neuroscience research, future directions should focus on enhancing their maturation and functionality. The progression of neural organoids approaches will generate more accurate and comprehensive disease models, ultimately adding to our understanding of disease pathogenesis and paving the way for future precision therapies for neurological diseases.

## Introduction

Human pluripotent stem cells (hPSCs), with their remarkable ability to self-renew and replicate indefinitely in culture while maintaining their undifferentiated state, have become invaluable tools in disease modeling studies. These cells can differentiate into any cell type, making them particularly useful for studying neurodevelopmental and neurodegenerative diseases. The groundbreaking work of Yamanaka, who developed induced pluripotent stem cells (iPSCs) from adult cells, opened up new possibilities for studying patient-specific cells with unique genetic backgrounds (Takahashi and Yamanaka, 2006). hPSCs have been shown to differentiate into various cell types, such as midbrain dopamine neurons and cortical neurons, facilitating research into diseases such as Parkinson’s disease (PD), Alzheimer’s disease (AD), and neurodevelopmental disorders (NDDs) (Kriks et al., 2011; Chen et al., 2015). This method addresses the limitations of animal models, which often fail to accurately mimic human pathogenesis (Jucker, 2010; Huch et al., 2017; Chandra Prakash, 2024), and the ethical concerns associated with human tissue usage (Council, 2014; Ananthamurthy, 2016).

Given the complexity of neurological diseases, two-dimensional (2D) cultures derived from hPSCs may not suffice for comprehensive disease modeling. 2D cultures, consisting of a single pure cell type, cannot recapitulate the actual complexity and spatial organization of the brain. Neural organoids (NOs), three-dimensional (3D) structures derived from hPSCs, have emerged as a superior alternative to traditional models such as animal studies and 2D cultures. These 3D structures closely mimic the cellular diversity and architecture of the human brain, providing a more physiologically relevant platform for research. Furthermore, the ability to create personalized NOs from patient-derived iPSCs allows for the investigation of individual-specific disease mechanisms and responses to treatments, leading to more targeted and effective therapeutic strategies (Lancaster et al., 2013; Quadrato et al., 2016; Pașca et al., 2019).

In this review, a comprehensive overview of the current state of NOs research is provided. The current review aims to summarize existing protocols for various types of NOs, emphasizing the key growth factors involved in their patterning and development. Additionally, it explores the potential use of co-culture techniques, where NOs are combined with different cell types or organoids to create more complex and physiologically accurate models. By highlighting these advancements and addressing the challenges in NOs research, this review seeks to shed light on the future directions and translational applications of these models in neuroscience, underscoring their potential to transform the field.

## Search Strategy

A comprehensive literature search was performed using PubMed, Scopus, and Web of Science databases via their respective institutional platforms. Searches covered publications from January 2000 through June 2025 to capture the emergence and progression of neural organoid technologies. The following combinations of keywords and Medical Subject Headings (MeSH) terms were used: “neural organoid” OR “brain organoid” OR “cerebral organoid” OR “midbrain organoid” OR “cortical organoid” OR “hippocampus organoid” OR “diencephalon organoid” OR “hypothalamus organoid” OR “thalamus organoid” OR “spinal cord organoid” OR “cerebellar organoid” OR “assembloid” AND “disease modeling” OR “neurodevelopmental disorders” OR “psychiatric disorders” OR “neurodegenerative disorders” OR “neuroinfections” OR “drug screening.” No language restrictions were applied, but only peer-reviewed original articles and reviews were considered. Reference lists of relevant articles were also screened to identify additional studies.

## Neural Organoid Approaches

NOs are powerful, region-specific models of the human central nervous system (CNS), each tailored to recapitulate distinct aspects of brain development, function, or disease. Generation of NOs begins with embryoid bodies (EBs), which are then directed toward neural fates by timed exposure to patterning factors. EBs can be produced by two principal methods, including spontaneous aggregation and forced aggregation (Pesl et al., 2014; Pettinato et al., 2015). In the spontaneous aggregation protocol, hPSCs are cultured in non-adherent conditions, such as low-attachment plates or suspension cultures, where they self-organize into EBs (Pettinato et al., 2015). Although this protocol mimics natural cell-cell interactions, it often yields EBs with variable size and morphology (Pettinato et al., 2015). By contrast, the forced aggregation technique confines cells within defined microstructures (e.g., microwells or microtubes), driving uniform aggregation and producing EBs of consistent diameter and shape, which enhances reproducibility and standardization (Pesl et al., 2014).

**Figure 1:**
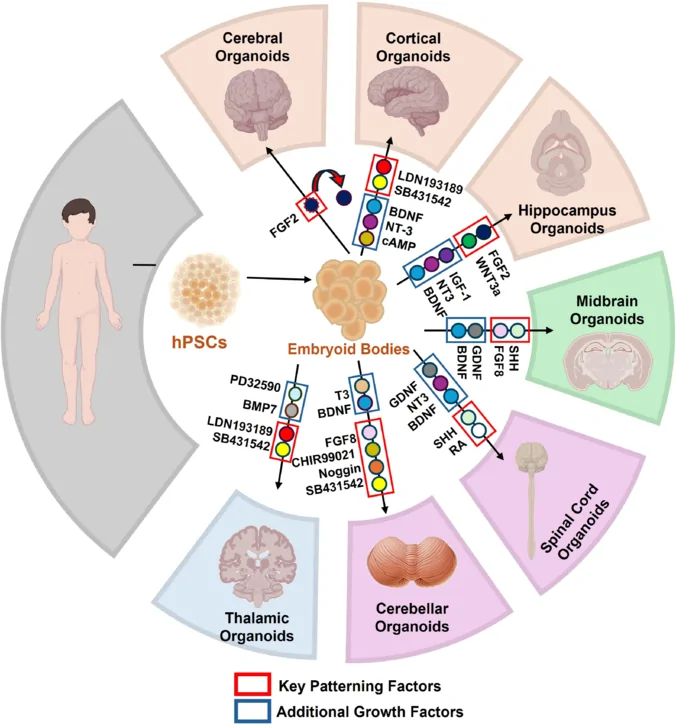
Overview of neural organoids protocol and patterning from human pluripotent stem cells. The differentiation of hPSCs into various types of neural organoids is guided by different growth factors, indicated by the colored dots in the legend. Factors in the red box are the key patterning factors for differentiation of specific types of organoids, while factors in the blue box are the supplementary growth factors to ensure cell fate, growth, and survival. Created with BioRender.com. SB431542: Transforming growth factor-beta receptor inhibitor; BDNF: brain-derived neurotrophic factor; BMP7: bone morphogenetic protein 7; cAMP: cyclic adenosine monophosphate; CHIR99021: GSK3 inhibitor; FGF2: fibroblast growth factor 2; FGF8: fibroblast growth factor 8; GDNF: glial cell–derived neurotrophic factor; hPSCs: human pluripotent stem cells; IGF-1: insulin-like growth factor 1; LDN193189: bone morphogenetic protein inhibitor; Noggin: bone morphogenetic protein inhibitor; NT-3/NT3: neurotrophin-3; RA: retinoic acid; SHH: sonic hedgehog; T3: triiodothyronine; WNT3a: Wnt family member 3A.

Following EB formation, region-specific patterning cues guide differentiation into three major classes of neural organoids, classified according to the region of the CNS they model (**Figure 1**): (1) forebrain organoids, which encompass telencephalon-derived models such as cerebral organoids (COs) generated via unguided protocols, as well as regionally specified cortical (CtOs), hippocampal (HOs), and ventral telencephalon organoids (e.g., medial ganglionic eminence and striatal models), alongside diencephalic derivatives such as hypothalamic and thalamic organoids (ThOs); (2) midbrain organoids (MOs), which recapitulate mesencephalic development and dopaminergic neuron specification; and (3) hindbrain and spinal cord organoids, encompassing cerebellar (CerOs) and spinal cord organoids (SCOs). This classification, grounded in developmental neuroanatomy, provides a clearer framework for understanding the diversity of NO systems and their applications in modeling human CNS development and disease. The neuralized and patternized EBs were embedded in Matrigel, which provided structural support and facilitated radial glia formation and neuroepithelial organization. To create a 3D culturing environment for these organoids, the cells are cultured in a spinning bioreactor to enhance nutrient and oxygen supply, mimicking the dynamic environment of the developing brain.

### Forebrain organoids

Forebrain organoids are classified into two different groups: unguided differentiation for COs generation, and guided differentiation for CtOs and HOs generation.

#### Cerebral organoids

COs are types of 3D cultured NOs that recapitulate the overall structure of the human brain. These organoids contain various brain regions, including the cortex, and are used to study general brain development and diseases. Generation of these organoids is unguided forebrain-derived organoids. Several studies have explored the creation and development of COs, a field pioneered by Lancaster et al. in 2013. Following this pioneering work, other studies by Qian et al. (2016) and Giandomenico et al. (2019) have developed COs to study Zika virus infection and created cultured COs at the air-liquid interface to generate diverse nerve tracts with functional output, enhancing the physiological relevance of organoids and their potential for studying brain function and diseases.

In addition to structural features, the functional activity of COs has been demonstrated, highlighting their ability to replicate critical aspects of brain physiology. Electrophysiological studies, such as calcium imaging and multi-electrode arrays, have shown that COs possess functional circuit architecture capable of generating diverse spiking patterns. These findings indicate the presence of electrically active neurons forming synaptic connections (Sharf et al., 2022).

EBs from hPSCs were cultured in neural induction medium without exogenous morphogens, allowing spontaneous neuroectoderm differentiation. After a few days, this protocol allowed the organoids to grow and differentiate without forced regionalization, instead relying on intrinsic self-patterning mechanisms to mimic early human brain development. The generation of COs typically takes 3–6 months to mature, with some protocols even extending up to a year for more complex structures (Lancaster and Knoblich, 2014). COs often display a lack of consistent cortical lamination and show limited long-term viability in the core due to absent vasculature, which constrains maturation and the modeling of late-onset phenotypes.

#### Cortical organoids

Other forebrain organoid types include CtOs, which specifically model the cerebral cortex, the region responsible for higher cognitive functions. In contrast to COs, CtOs are forebrain-derived structures generated through time-dependent morphogen guidance. CtOs are utilized to study cortical development and disorders such as autism and schizophrenia (SCZ) (Paşca et al., 2015). Recent studies have advanced our understanding of cortical development by employing various innovative approaches to model human cortical development and associated disorders. Pasca et al. (2015) focused on the assembly of functional cortical neurons and astrocytes in a 3D culture environment, creating a robust platform for studying human cortical development and neurodevelopmental disorders. This work provided significant insights into the differentiation and maturation processes of cortical cells. Building on these foundations, Winden et al. (2025) utilized CtOs to model malformations of cortical development, highlighting their utility in studying neurodevelopmental disorders such as epilepsy and autism spectrum disorder (ASD).

Furthermore, CtOs have been shown to exhibit functional characteristics of neuronal activity, including complex oscillatory waves that mirror early stages of human brain network development. Recent studies have employed electrophysiological techniques such as calcium imaging and multi-electrode arrays, which demonstrate coordinated neuronal firing and the formation of functional circuit architectures. These findings highlight the ability of CtOs to recapitulate key developmental processes, such as synaptic plasticity and connectivity, that are crucial for modeling human brain function and disorders (Trujillo et al., 2019). Zourray et al. (2022) reviewed the electrophysiological properties and functional maturation of neurons within human CtOs, offering a comprehensive overview of their electrophysiological characterization.

The CtOs establishment protocol is specifically designed to model the cerebral cortex and involves the differentiation of stem cells into cortical neurons. Similar to COs, CtOs are generated from EBs, which are cultured in a defined medium. A crucial step in the differentiation process is the use of dual-SMAD inhibitors (dorsomorphin and SB431542), which effectively induce neural differentiation by inhibiting both bone morphogenetic protein (BMP) and transforming growth factor-β signaling pathways (Paşca et al., 2015). This inhibition ensures the development of a robust and lasting cortical neural stem cell identity.

The patterning and development of cortical neurons are further promoted by the supplementation of specific growth factors, such as brain-derived neurotrophic factor (BDNF), neurotrophin-3 (NT-3), and cyclic adenosine monophosphate, which support neuronal growth and differentiation. The presence of these growth factors is vital for the proliferation and maturation of cortical neurons, leading to the formation of complex and physiologically relevant CtOs. The generation of cortical organoids typically takes 60–80 days to achieve a mature structure (Paşca et al., 2015). CtOs can recapitulate early cortical layer markers but frequently show incomplete or inconsistent laminar organization and variable proportions of outer radial glia, limiting replication of later-stage cortical circuit maturation and reliable electrophysiological benchmarks.

#### Hippocampal organoids

HOs model the hippocampus, a region critical for memory and learning, and are used to study neurodegenerative diseases such as AD. Several studies have developed HOs using unique approaches to advance our understanding of hippocampal development and function. These studies have demonstrated the formation of regionalized dorsomedial telencephalic tissue, modeled SCZ by focusing on the pathology and dysfunction of hippocampal neurons. HOs are generated via guided forebrain differentiation protocols and its functionality has been validated by testing their spike activity, which revealed the presence of electrically active neurons capable of generating coordinated firing patterns and forming functional circuits. Other studies investigated mechanisms of early hippocampal development, activity-dependent gene expression, and circuit formation (Sakaguchi et al., 2015; Pomeshchik et al., 2020). Collectively, these studies highlight the diverse methodologies and contributions to the field, offering valuable insights into the development, function, and pathology of the hippocampus.

The formation of HOs involves specific growth factors and signaling pathways to induce neural induction and hippocampal patterning. EBs from hPSCs are treated with WNT3a and fibroblast growth factor 2 (FGF2) to promote hippocampal differentiation. WNT3a and FGF2 are critical for initiating the differentiation process, guiding the cells towards a hippocampal identity (Sakaguchi et al., 2015).

To further enhance hippocampal neuron growth and maturation, specific growth factors such as BDNF, NT-3, and insulin-like growth factor 1 are added to the culture. These growth factors are essential for supporting the proliferation, differentiation, and survival of hippocampal neurons, ensuring the development of a complex and physiologically relevant hippocampal structure (Xiang et al., 2017). The generation of HOs typically spans 60–80 days, with the duration depending on the specific protocol and desired level of maturity (Sakaguchi et al., 2015). Compared with other organoid models, HOs commonly fail to produce well-demarcated CA and dentate gyrus subfields and lack mature trisynaptic circuitry. As a result, cell-type diversity, laminar organization, and functional readouts that depend on mature hippocampal circuitry remain limited, constraining their utility for modeling memory-dependent disease phenotypes until protocols achieve more complete maturation.

### Thalamic organoids

ThOs mimic the thalamus, which acts as a relay station for sensory and motor signals in the brain, and are used to study sensory processing and related disorders. Several studies have developed ThOs to advance our understanding of thalamic development and function. Protocols have been established to generate regionally specified NOs from hPSCs, recapitulating thalamus development to study related diseases and discover potential therapeutics. Notably, the functionality of ThOs has been validated by highlighting their ability to model thalamic network activity and validate physiological relevance. Generation of ventralized ThOs containing distinct subregions, including the thalamic reticular nucleus, and demonstrated functional connectivity through electrophysiological assessments, which confirmed the presence of synaptic activity and neural interactions (Kiral et al., 2023). Recent progress includes constructing brain organoids with subregional identity, including the thalamus, by applying specific signaling pathways and neurotrophic factors to guide stem cell differentiation (Xiang et al., 2020a; Xiang and Park, 2024). Collectively, these studies highlight the diverse methodologies and contributions to the field, offering valuable insights into the development, function, and pathology of the thalamus.

ThOs are generated from EBs that are maintained in a defined neural induction medium supplemented with the dual-SMAD pathway inhibitors SB431542 (transforming growth factor-β inhibitor) and LDN-193189 (BMP inhibitor), which synergistically suppress mesodermal and endodermal fates while promoting neuroectodermal specification. The medium also contains the ROCK inhibitor Y-27632 during the initial four days to enhance cell survival during aggregation. By day 4, Y-27632 is withdrawn, allowing the cells to stabilize and consolidate a neural progenitor identity, which is typically well established by day 8 (Kiral et al., 2023).

Following neural induction, the developing organoids are transferred to larger ultra-low-attachment 6-well plates to accommodate growth and to initiate regional patterning. From days 8 to 16, the cultures are maintained in a minus vitamin A medium containing BMP7 and the mitogen-activated protein kinase kinase/extracellular-signal-regulated kinase pathway inhibitor PD325901. This combination is critical for steering the tissue toward a default thalamic identity by mimicking aspects of diencephalic developmental signaling while preventing alternative forebrain fates (Kiral et al., 2023).

After the patterning phase, the organoids are transitioned into a differentiation medium enriched with BDNF and ascorbic acid to support neuronal survival, maturation, and synaptic development. Over-extended culture periods, often beyond day 70, this protocol yields organoids that express canonical dorsal thalamic transcription factors such as LHX2 and LHX9, and that predominantly generate glutamatergic excitatory neurons resembling those found in the dorsal thalamus. These ThOs serve as a baseline model for comparison with ventralized thalamic organoids (vThOs), which undergo additional late-stage sonic hedgehog (SHH) treatment to induce ventral thalamic identities. Diencephalic models may capture regional markers but typically lack precise subnuclear organization and afferent–efferent connectivity, making it difficult to study complex thalamocortical relay function or hypothalamic neuroendocrine circuitry at scale (Kiral et al., 2023).

### Midbrain organoids

MOs focus on the midbrain region, involved in motor control and sensory processing, making them particularly useful for studying diseases such as PD (Jo et al., 2016). Several studies have focused on the development of MOs, each employing unique approaches to advance our understanding of brain development and disease modeling. The first attempt to create midbrain-like organoids from hPSCs was undertaken by Jo et al. (2016), resulting in the generation of functional dopaminergic and neuromelanin-producing neurons. Notably, Jo et al. (2016) also validated the functionality of these organoids by demonstrating electrically active dopaminergic (DA) neurons capable of generating action potentials and forming synaptic connections. Furthermore, these organoids secreted dopamine and exhibited neuromelanin-like granules, mirroring key pathological features of the human midbrain, particularly in the context of PD (Jo et al., 2016).

Other studies have also been shown to developed standardized protocol, evaluate the region specific marker over time, and to create MOs containing abundant DA neurons, astroglia, and oligodendrocytes, demonstrating their potential for modeling PD by comparing organoids with disease-specific mutations to healthy controls (Smits and Schwamborn, 2020; Mohamed et al., 2021; Alam El Din et al., 2024). Collectively, these studies highlight the diverse methodologies and contributions to the field, offering valuable insights into the development, function, and pathology of the midbrain.

A critical step in inducing neural development is the treatment of these EBs with SHH and fibroblast growth factor 8 (FGF8), which are essential for establishing ventral midbrain identity (Roussa and Krieglstein, 2004).

The midbrain patterning is further refined by the addition of glial cell line-derived neurotrophic factor (GDNF) and BDNF, which support the survival and maturation of DA neurons. GDNF, in particular, plays a vital role in promoting the survival of DA neurons, ensuring their proper function and longevity (Yeap et al., 2023). The generation of MOs typically takes 50–70 days to achieve maturity (Jo et al., 2016). Although MOs generate dopaminergic neurons, they often exhibit immature metabolic and electrophysiological profiles, variable dopaminergic yields across lines, and limited oligodendrocyte/myelination development, all of which constrain modeling of age-dependent Parkinsonian phenotypes.

### Hindbrain and spinal cord organoids

#### Cerebellar organoids

CerOs mimic the cerebellum, a region located at the back of the brain that contains the highest number of neurons in the CNS. The cerebellum connects the brain to the spinal cord and plays a crucial role in motor coordination and cognitive functions. It is essential for voluntary muscle movement and is increasingly recognized for its involvement in higher-order brain processes (Jimsheleishvili and Dididze, 2023). CerOs are valuable models for studying neurodegenerative diseases such as spinocerebellar ataxias and Friedreich’s ataxia (FRDA). They also provide insights into neuronal network formation and the development of cerebellar circuits, offering a deeper understanding of cerebellar function in both health and disease (Chen et al., 2023; Atamian et al., 2024a).

These organoids are generated by inducing midbrain-hindbrain patterning using a specific set of morphogens. These include SB431542, Noggin, CHIR99021, and FGF8b. During differentiation, the cells organize into structures resembling ventricular zones and rhombic lips—key features of cerebellar development that help establish a regional identity similar to that of human cerebellar tissue.

Further maturation steps involve supplementing the culture with triiodothyronine and BDNF. These factors promote the development and maturation of key cerebellar cell types, including Purkinje cells, granule neurons, and inhibitory interneurons. The resulting organoids exhibit functional synaptic connectivity, making them a powerful platform for investigating cerebellar disorders and testing therapeutic compounds (Atamian et al., 2024b). CerOs can produce key cerebellar cell types yet show protracted maturation, low Purkinje cell yield/maturation, and incomplete circuit assembly, hindering studies that require mature cerebellar physiology and motor learning readouts.

#### Spinal cord organoids

SCOs replicate the spinal cord and are crucial for studying spinal cord development and diseases such as amyotrophic lateral sclerosis (ALS). Multiple studies have developed SCOs using different approaches to advance our understanding of spinal cord development, disease modeling, and regenerative medicine. For instance, the formation of SCOs with diverse cell types, including motor neurons, interneurons, and astrocytes, has been demonstrated (Hor et al., 2018). Notably, the studies by Lee et al. (2022) validated the functionality of SCOs by demonstrating their ability to exhibit mature synaptic activities, alongside neural-tube morphogenesis. This validation underscores their physiological relevance and utility for studying neurodevelopment and associated disorders (Lee et al., 2022). Additionally, other studies have improved regional specification and cellular diversity, enhanced the predictive ability for drug screening, and explored the use of different embedding matrices such as alginate to create SCOs that can mimic pathological conditions (Iyer and Ashton, 2022; Chooi et al., 2023; Ruiqi et al., 2024). Collectively, these studies provide valuable insights into spinal cord development, function, and pathology.

A critical step in spinal cord patterning is the treatment of these cells with appropriate concentrations of CHIR99021, a GSK-3 inhibitor, and retinoic acid, which induces caudalization. These factors are essential for neural differentiation and establishing spinal cord identity. Often, SHH agonists are added to promote ventralization, enriching the organoids with motor neurons for studying motor neuron development and associated disorders (Ogura et al., 2018).

To further enhance spinal cord neuron growth, specific growth factors such as BDNF, NT-3, and GDNF are added to the culture. GDNF, in particular, plays a vital role in promoting the survival and maturation of spinal cord neurons, ensuring their proper function and longevity (Deng et al., 2011). The generation of SCOs typically takes 40–60 days, with the duration varying depending on the specific protocol and desired level of maturity (Hor et al., 2018; Ogura et al., 2018; Iyer and Ashton, 2022; Chooi et al., 2023; Ruiqi et al., 2024). SCOs reproduce ventral/dorsal patterning and motor neuron populations but generally lack fully functional neuromuscular junction maturation and peripheral cell types unless paired with muscle compartments, which reduces translational fidelity for motor disorders.

Each type of NOs generation follows a distinct protocol involving specific patterning, growth factors, and differentiation durations, as summarized in a comparative table (**Table 1**).

**Table 1:** Summary of neuronal organoid approaches

Neuronal organoid types	Key factors	Key patterning steps	Additional growth factors	Durations	References
COs	3D self organization	Pluripotency factors removal, neural induction medium	NA	3–6 mon	Lancaster and Knoblich, 2014
CtOs	Dorsomorphin (BMP inhibitor), SB431542 (transforming growth factor-β inhibitor)	Dual-SMAD inhibitor to induce cortical neural stem cell identity	BDNF, NT-3, cAMP	50–70 d	Paşca et al., 2015
HOs	WNT3a, FGF2	Induce hippocampal identity	BDNF, NT-3, insulin-like growth factor 1	60–80 d	Sakaguchi et al., 2015
ThOs	Dual-SMAD inhibitors (SB431542 and LDN-193189)	BMP7 and the MEK-ERK inhibitor PD32590	BDNF, ascorbic acid	70–110 d	Kiral et al., 2023
MOs	SHH, FGF8	Induce ventral midbrain identity	GDNF (Dopamine neuron survival), BDNF	50–70 d	Jo et al., 2016
CerOs	SB431542, Noggin, CHIR99021 FGF8b	Induce midbrain-hindbrain to organize into ventricular zones and rhombic lips	Triiodothyronine and BDNF	30–60 d	Atamian et al., 2024
SCOs	Retinoic acid, SHH	Induce caudalization and ventralization	BDNF, NT-3, GDNF	60–80 d	Ogura et al., 2018

3D: Three-dimensional; BDNF: brain-derived neurotrophic factor; BMP: bone morphogenetic protein; cAMP: cyclic adenosine monophosphate; CerOs: cerebellar organoids; COs: cerebral organoids; CtOs: cortical organoids; ERK: extracellular-signal-regulated kinase; FGF: fibroblast growth factor; GDNF: glial cell line-derived neurotrophic factor; HOs: hippocampal organoids; MEK: mitogen-activated protein kinase kinase; MOs: midbrain organoids; NA: not available; NT-3: neurotrophin-3; SCOs: spinal cord organoids; SHH: sonic hedgehog; ThOs: thalamic organoids.

## Neural Organoid Approaches for Disease Modeling

Organoid models have significantly advanced our understanding of various neurological diseases and developmental processes. This section focuses on NO models that have played a crucial role in elucidating disease mechanisms, particularly those with strong genetic associations. Priority was given to studies utilizing patient-derived iPSCs, which enable the identification of disease-specific mutations and their effects on neural development. We included models that accurately recapitulate key pathological features of neurological disorders, such as disrupted neural progenitor dynamics, altered synaptic connectivity, and protein aggregation. Specifically, cerebral, cortical, midbrain, spinal cord, thalamic, and hippocampal organoids have been pivotal in uncovering disease mechanisms, testing potential therapies, and advancing personalized medicine.

### Neurodevelopmental disorders

Neurodevelopmental disorders such as autism spectrum disorders, microcephaly, Rett syndrome, Timothy syndrome, and tuberous sclerosis arise from disruptions in early brain development, and neural organoids provide a powerful platform to model progenitor dynamics, neuronal differentiation, and circuit formation that are inaccessible *in vivo.*

#### Microencephaly

Organoids have been instrumental in modeling brain development and neurodevelopmental disorders. Notable achievements include the use of iPSCs to generate COs by Lancaster et al. in 2013 to study the organization of progenitor zones with abundant outer radial glial cells. This model provides a more accurate representation of human brain development compared to traditional animal models. The use of RNAi to target the CDK5RAP2 gene, which is involved in underdeveloped brains, successfully mimics microcephaly conditions in cerebral organoids generated from patient-derived iPSCs (Lancaster et al., 2013).

#### Autism spectrum disorder

Organoids such as CtOs have also provided significant insights into cortical development and NDD, such as autism. A study by Mariani et al. showed that the specific iPSCs derived CtOs in ASD patients have an increase in expression of FOXG1 genes, leading to the overproduction of GABAergic neurons in these organoids. This dysregulation contributes to the imbalance between excitatory and inhibitory neurons, which is a hallmark of ASD, showing that FOXG1 might be a potential target for understanding and treating ASD (Mariani et al., 2015).

A study by Urresti et al. in 2020 showed the importance of 16p11.2 copy number variants (CNVs) using CtOs from skin fibroblasts of patients with 16p11.2 CNVs, which is the most common CNV in ASD. They found that this CNV alters the ratio of neurons to neural progenitors during early neurogenesis, leading to an abnormal size of the brain. The study identified synaptic defects and neuron migration as key drivers of functional effects of the 16p11.2 CNV, and inhibition of the small GTPase RhoA activity rescued migration deficits, implicating RhoA upregulation as one of the pathways impacted by the 16p11.2 CNV (Urresti et al., 2021).

#### Rett syndrome

A recent study in 2020 by Xiang et al. demonstrated that MeCP2-mutant human CtOs recapitulate Rett syndrome (RTT)-specific interneuron deficits. Mechanistically, aberrant genome-wide binding of BRD4 in mutant organoids drives persistent transcriptional hyperactivity, disrupting neurodevelopment and impairing neuronal activity/plasticity. Crucially, low-dose JQ1 (a BET bromodomain inhibitor) rescued BRD4-mediated chromatin dysregulation, normalized transcriptional programs, and reversed neurodevelopmental abnormalities. These findings identify BRD4 dysregulation as a central pathogenic mechanism in RTT and propose BRD4 inhibition as a promising therapeutic strategy for RTT (Xiang et al., 2020b).

#### Timothy syndrome

Another study by Birey et al. in 2017 revealed that another NDDs—Timothy syndrome (TS) with the clinical features such as cognitive impairment and autism, caused by CACNA1C mutations disrupting CaV1.2 L-type calcium channel function, exhibits impaired cortical interneuron migration in human assembloid models. By fusing patient-derived pallial and subpallial CtOs, researchers observed aberrant interneuron migration characterized by increased saltation frequency but reduced displacement length and speed, indicating compromised cortical circuit development. Critically, pharmacological L-type calcium channel inhibition rescued these migratory deficits, highlighting CaV1.2 dysregulation as a key pathological mechanism and proposing therapeutic potential for calcium channel modulation in TS-related neurodevelopmental abnormalities (Birey et al., 2017).

A recent study by Chen et al. in 2024 also revealed important findings in TS about TS type 1 (TS1), which is driven by a gain-of-function mutation in the developmentally regulated CACNA1C exon 8A, resulting from neuronal dysregulation and persistent exon 8A expression. To counteract this, antisense oligonucleotides (ASOs) were designed to suppress exon 8A inclusion and promote splicing of exon 8, effectively normalizing CACNA1C isoform balance. In patient-derived CtOs, ASO treatment rescued calcium dyshomeostasis, interneuron migration deficits, and dendrite retraction. Notably, intrathecal ASO delivery *in vivo* restored physiological calcium dynamics and dendritic integrity in transplanted patient neurons. These findings establish exon switching via ASOs as a promising therapeutic strategy for TS (Chen et al., 2024).

#### Tuberous sclerosis complex

A study by Blair et al. in 2018 elucidated the pathogenesis of tuberous sclerosis complex (TSC), NDDs caused by biallelic mutations in TSC1/TSC2 (encoding mTORC1 inhibitors hamartin/tuberin), leading to mTORC1 hyperactivation and cortical tuber formation—dysplastic cortical regions with disorganized neurons and glia in mutant organoids. This work highlights the necessity of human organoid models, as cortical tubers, a hallmark of TSC, are human-specific and absent in murine models. Pharmacological mTORC1 inhibition with rapamycin markedly reduced tuber formation when administered early in organoid development, highlighting the therapeutic potential in TSC (Blair et al., 2018).

Another recent study by Eichmüller et al. (2022) also established the human organoid model of TSC and newly identified caudal late interneuron progenitor cells—a neural stem cell population whose hyperproliferation drives TSC key pathogenesis. They found that caudal late interneuron progenitor overexpansion leads to excessive interneuron production, cortical malformations, and brain tumorigenesis in TSC. Pharmacological inhibition of epidermal growth factor receptor signaling effectively reduces tumor burden, highlighting epidermal growth factor receptor as a therapeutic target for TSC and related disorders (Eichmüller et al., 2022).

#### Focal cortical dysplasia

Recent work in 2025 by Xu et al. highlights the role of patient-derived region-specific organoids in mirroring the pathogenic mechanisms of focal cortical dysplasia, an NDD marked by epilepsy-associated cortical malformations. The pathological features, including dysmorphic neurons, balloon cells, astrogliosis, ectopic cardiomyocyte-like cells, and propagative neuronal hyperactivity, are linked to sustained BMP/WNT signaling activation. Notably, the aberrant presence of cardiomyocyte-like cells in CtOs suggests their potential heterotopic origin and transformation into malformed neurons, which may drive circuit hyperexcitability in focal cortical dysplasia. These findings propose that cardiomyocyte heterotopia and the propagation of neuronal hyperactivity represent novel therapeutic targets for focal cortical dysplasia (Xu et al., 2025).

#### Other neurodevelopmental disorders

Organoids, such as sliced CtOs, can be used to model distinct cortical layer formation, maintaining cell viability and sustaining growth, as shown by a study by Qian et al. in 2020. This study reveals the WNT/β-catenin signaling pathway as a key regulator of neuronal fate specification within the cortical layers. They also observed that mutations in specific genes, such as DISC1, caused deficits in cortical neuron fate specification, highlighting the importance of these pathways in cortical development. This study provides a valuable method for generating and studying human CtOs, offering insights into cortical layer formation and the molecular mechanisms underlying cortical development (Pereira et al., 2021).

### Neurodegenerative disease

Organoids have been proven to be a good platform to model neurodegenerative disorders, including Parkinson’s disease, Alzheimer’s disease, Huntington’s disease, amyotrophic lateral sclerosis, and spinocerebellar ataxias, which are marked by progressive neuronal loss, and organoids provide human-relevant systems to investigate protein aggregation, mitochondrial dysfunction, and selective neuronal vulnerability.

#### Alzheimer’s disease

COs have emerged as powerful platforms for studying AD, particularly in individuals with Down syndrome (Trisomy 21, T21), who carry an extra copy of the amyloid beta precursor protein (*APP* gene). This gene duplication leads to elevated amyloid-β (Aβ) production, increasing the risk of early-onset AD. A study by Alić et al. (2021) demonstrated that T21-derived COs display hallmark AD-like pathology, including extracellular Aβ deposits, hyperphosphorylated tau (pTau), and premature neuronal loss. Interestingly, BACE2, another gene located on chromosome 21, was found to play a protective role by reducing Aβ aggregation. When the third copy of BACE2 was removed via CRISPR-Cas9 editing, AD pathology worsened, confirming its dose-sensitive suppressor function (Alić et al., 2021). Supporting this, a separate study using CtOs from a Hirschsprung’s disease patient with BACE2 haploinsufficiency also demonstrated increased Aβ pathology, further reinforcing neuroprotective role of BACE2 (Luo et al., 2022). Additionally, analysis of conditioned media from T21-derived COs revealed elevated levels of soluble Aβ and pTau aggregates—key drivers of AD pathology. Notably, these aggregates were detectable before the appearance of histological amyloid plaques or intraneuronal tau pathology, suggesting that early-stage AD mechanisms can be studied non-invasively using this model (Alić et al., 2021; Fertan et al., 2024). Collectively, these findings highlight the utility of COs in unraveling the molecular underpinnings of AD in T21 and support their application in developing precision medicine approaches aimed at delaying or preventing neurodegeneration.

HOs have also shown great promise in modeling AD and other neurodegenerative disorders. Pomeshchik et al. (2020) developed human iPSC-derived hippocampal spheroids as an innovative tool for stratifying AD patient-specific cellular phenotypes and developing therapies. They established a protocol to efficiently and rapidly generate hippocampal neurons from hiPSCs. These hippocampal spheroids, derived from AD patients carrying APP or presenilin 1 (*PS1*) gene variations, exhibited key pathological features of AD. They were used to model the disease, revealing increased Aβ_42_/Aβ_40_ peptide ratios and decreased synaptic protein levels in patient-specific spheroids. The study also explored gene therapy strategies to modulate the expression of synaptic transmission-related genes, providing a valuable platform for targeted AD therapies (Pomeshchik et al., 2020).

#### Parkinson’s disease

MOs have proven to be particularly valuable for studying PD, a neurodegenerative disorder characterized by the loss of dopaminergic neurons in the substantia nigra. Jo et al. (2016) successfully created human MOs (hMOs) containing functional dopaminergic neurons and neuromelanin-producing neurons. These hMOs exhibited electrically active and functionally mature midbrain DA neurons, which not only produced and secreted dopamine but also formed neuromelanin-like granules structurally similar to those found in human substantia nigra tissues—a hallmark of PD pathology (Jo et al., 2016). This groundbreaking work established hMOs as a robust model for studying PD.

Further advancing this field, Jo et al. (2021) demonstrated that hMOs derived from hPSCs carrying mutations in the glucocerebrosidase (*GBA1*) and α-synuclein (*α-syn*) genes exhibited a substantial accumulation of detergent-resistant, β-sheet–rich α-syn aggregates and Lewy body–like inclusions. These inclusions, which are characteristic of PD, displayed a spherically symmetric morphology with an eosinophilic core and contained α-syn and ubiquitin. Importantly, similar Lewy body–like inclusions were also observed in hMOs derived from PD patients, providing strong evidence that loss of glucocerebrosidase function promotes Lewy body formation. This study offers a tractable model for elucidating the mechanisms underlying progressive Lewy body formation, shedding light on the pathogenesis of PD and identifying potential therapeutic targets (Jo et al., 2021).

In another significant study, Kim et al. (2019) generated MOs from iPSCs of patients carrying the LRRK2-G2019S mutation, a common genetic risk factor for PD. These organoids recapitulated key pathological hallmarks of PD, including reduced numbers and complexity of midbrain DA neurons. Additionally, the study identified TXNIP, a thiol-oxidoreductase, as a critical player in the development of LRRK2-associated PD within the 3D organoid environment (Kim et al., 2019). These findings highlight the utility of MOs in understanding the mechanisms of PD and provide a platform for investigating the role of genetic mutations in disease progression.

Collectively, these studies underscore the potential of MOs as a powerful tool for modeling PD, elucidating disease mechanisms, and identifying novel therapeutic targets. By recapitulating key pathological features of PD, such as Lewy body formation and dopaminergic neuron loss, MOs offer a promising avenue for advancing our understanding of this debilitating disease and developing targeted therapies.

#### Amyotrophic lateral sclerosis

SCOs have become crucial tools for understanding spinal cord development and modeling diseases such as ALS. A previous study by Guo et al. in 2023 demonstrated that SCOs derived from C9orf72 knockdown human iPSCs recapitulated key features of ALS, including the upregulation of pro-inflammatory factors. This finding highlights the importance of neuroinflammation in ALS pathology and underscores the utility of organoids in studying disease mechanisms (Guo et al., 2024).

Because distal denervation and neuromuscular junctions (NMJ) dysfunction are hallmarks of ALS, researchers have extended SCO protocols to create neuromuscular organoids (NMOs), which combine a spinal-cord domain with a mesodermal compartment that differentiates into skeletal muscle fibers and supportive Schwann cells. Using NMOs derived from C9orf72 patient iPSCs, Gao et al. (2024) observed denervated NMJs, decreased contractile strength, and Schwann-cell loss, in addition to neuronal hallmarks of ALS such as RNA foci and dipeptide repeat proteins in both neurons and astrocytes (Gao et al., 2024). Importantly, when organoids derived from ALS subjects or isogenic lines harboring familial ALS mutations were compared to controls, significant impairments in NMJ structure and function were observed (reduced muscle contraction frequency, altered synaptic morphology) (Pereira et al., 2021).

#### Spinal muscular atrophy

SCOs have also proven valuable for modeling spinal muscular atrophy (SMA). A study by Hor et al. in 2018 used SMA patient-derived iPSCs to generate SCOs that recapitulated key disease features, such as motor neuron degeneration. The researchers found that cell cycle inhibitors, specifically CDK4/6 inhibitors, could protect motor neurons from degeneration in the SMA organoid model. Additionally, they identified pathways uniquely activated in SMA motor neurons, including hyperactivated endoplasmic reticulum stress, neuronal hyperexcitability, and defective spliceosome function. These findings suggest that targeting cell cycle pathways may offer a promising therapeutic strategy for SMA (Hor et al., 2018).

#### Friedreich’s ataxia

CerOs have been utilized to model FRDA, an autosomal recessive neurodegenerative disorder characterized by progressive damage to the nervous system—particularly the spinal cord, peripheral nerves, and cerebellum—resulting in impaired muscle coordination (Williams and Jesus., 2023). A 2025 study by Ryu et al. employed CerOs derived from FRDA patients derived iPSCs, incorporating a glial induction strategy to enrich the organoids with Bergmann glia. The resulting organoids exhibited disease-specific phenotypes, including reduced expression of the mitochondrial enzyme aconitase 2, abnormal mitochondrial morphology, elevated reactive oxygen species production, and increased apoptosis. Notably, these pathological features correlated with the length of disease-associated GAA repeats. Importantly, the observed phenotypes could be ameliorated through both pharmacological interventions using histone deacetylase inhibitors and genetic correction via CRISPR-based approaches. These findings underscore the potential of CerOs to faithfully recapitulate FRDA pathology and serve as a platform for evaluating therapeutic interventions with clinical relevance (Ryu et al., 2025).

#### Machado-Joseph disease

In a separate study, CerOs were also used to model Machado-Joseph disease, also known as spinocerebellar ataxias type 3, a progressive neurodegenerative disorder caused by mutations in the ATXN3 gene, which leads to impaired motor control and coordination (Bettencourt and Lima, 2011). Brás et al. (2022) demonstrated that CerOs derived from Machado-Joseph disease patient iPSCs exhibited an increased number of ventricular-like zones—suggestive of impaired maturation—as well as the presence of ataxin-3-positive protein aggregates, consistent with hallmark pathological features of the disease. These results suggest that CerOs models not only mimic normal developmental processes but also faithfully reflect disease-specific alterations, making them valuable tools for pathophysiological studies and drug discovery in spinocerebellar ataxias (Brás et al., 2022).

### Psychiatric disorders

Psychiatric disorders such as schizophrenia, bipolar disorder, and major depressive disorder involve disruptions in neural connectivity and synaptic function, and organoid models enable the study of patient-specific developmental trajectories and excitatory–inhibitory imbalances that are difficult to capture in animals.

#### Schizophrenia

A study by Stachowiak et al. in 2017 showed the use of COs derived from SCZ patients. The study revealed that organoids from SCZ patients exhibited abnormal scattering of proliferating neural progenitor cells from the ventricular zones throughout the intermediate and cortical zones. This maldevelopment was linked to decreased intracortical connectivity and changes in the orientation and morphology of calretinin interneurons. Interestingly, nuclear fibroblast growth factor receptor 1 (nFGFR1) was abundantly expressed by developing subcortical cells but was depleted from neuronal committed cells of the cortical zone. Blocking FGFR1 with PD173074 reproduced the loss of nFGFR1 and cortical neuronal maturation in hESC-derived COs. The study provides valuable insights into the early developmental origins of SCZ and highlights the role of FGFR1 signaling in cortical maldevelopment (Stachowiak et al., 2017).

ThOs mimic the thalamus, acting as a relay station for sensory and motor signals in the brain. Thalamocortical organoids have enabled *in vitro* modeling of neuropsychiatric disorders associated with genetic mutations. Shin et al. in 2024 developed thalamocortical organoids, co-culture of ThOs and CtOs, from hPSCs to model the 22q11.2 microdeletion, which is associated with a significant genetic risk for psychiatric disorders, including SCZ. The study revealed widespread transcriptional dysregulation in thalamic neurons and glia, including elevated expression of forkhead box protein P2 (FOXP2), a transcription factor implicated in psychiatric disorders. They demonstrated that the 22q11.2 microdeletion leads to overgrowth of thalamic axons via overexpression of FOXP2, mediating the overgrowth of reciprocal corticothalamic axons. Dysregulation of FOXP2 led to downregulation of roundabout guidance receptor 2, an axon repulsive receptor important for thalamocortical axon pathfinding. This study highlights the potential of thalamocortical organoids and assembloids in advancing our understanding of neuropsychiatric disorders (Shin et al., 2024).

### Neuroinfection and inflammation

Neurotropic infections such as Zika virus, severe acute respiratory syndrome coronavirus 2, and enteroviruses, as well as inflammatory insults, can impair brain development and function, and organoids offer a tractable model to dissect host–pathogen interactions, barrier disruption, and neuroimmune responses.

COs have been used to model the effects of Zika virus infection by Qian et al. in 2016. They managed to generate forebrain-specific organoids from iPSCs, which were used as the target for Zika virus infection, revealing how the virus causes microcephaly by disrupting neural progenitor cells (Qian et al., 2016).

More recently, SCOs have been employed to study the neuropathogenesis of enterovirus-A71 (EV-A71) infections. A 2024 study by Chooi et al. demonstrated that EV-A71 preferentially infects motor neurons in SCOs, leading to neurodegeneration through ferroptosis, an iron-dependent form of cell death. The organoid model provided a physiologically relevant system that closely mimics human neural tissue, enabling detailed observation of viral infection and its effects on motor neurons. The study identified the selective tropism of EV-A71 for motor neurons over neural progenitors in both 2D culture and 3D SCOs. This work highlights the utility of SCOs in modeling human diseases and provides valuable insights into the mechanisms of EV-A71 neuropathogenesis, offering potential therapeutic strategies to mitigate its effects on the nervous system (Chooi et al., 2024).

Achievements in the study of NOs have significantly advanced our understanding of various neurological and neurodegenerative disorders, offering potential therapeutic insights. Each organoid type has been instrumental in modeling specific diseases, uncovering key findings, and identifying therapeutic targets. A detailed table (**Table 2**) has been created to summarize these achievements, highlighting the remarkable progress made and identifying areas for further exploration.

**Table 2:** Achievements using neuronal organoid approaches

Categories	Diseases	Organoid types	Key findings	Implications for therapy
Neurodevelopmental disorders	Microcephaly (Lancaster et al., 2013)	COs	Generated cerebral organoids from iPSCs to study progenitor zones with outer radial glial stem cells. RNAi targeting CDK5RAP2 gene mimics microcephaly conditions in patient-specific iPSCs	Provides a more accurate representation of human brain development and a model for studying microcephaly
	ASD (Mariani et al., 2015; Urresti et al., 2021)	CtOs	FOXG1 upregulation leads to overproduction of GABAergic neurons, leads to imbalance between excitatory and inhibitory neurons	FOXG1 as a potential therapeutic target
		CtOs	16p11.2 CNVs altered neuron-to-neural progenitor ratios, synaptic defects, neuron migration issues	Implicating RhoA upregulation as one of the pathways impacted by 16p11.2 CNV
	RTT (Xiang et al., 2020)	CtOs	Mutations in MeCP2 result in significant abnormalities in human interneurons	Targeting BRD4 may be a viable therapeutic option for RTT since RD4 dysregulation is a key factor in the etiology of RTT
	TS	CtOs	The mutation in the CACNA1C gene, which codes for the pore-forming subunit of LTCC Cav1.2, recapitulated the abnormal saltatory migration of interneurons observed in TS patients (Birey et al., 2017)	Indicating the role of LTCC function in human cortical interneuron migration and strategies to restore deficits of TS
		CtOs	TS1 driven by a gain-of-function mutation in the developmentally regulated CACNA1C exon 8A, results in neuronal dysregulation and can be rescued by antisense oligonucleotides-mediated switch from exon 8A to 8 (Chen et al., 2024)	The inhibition of CACNA1C exon 8A expression is a possible therapeutic approach for TS1
	TSC (Blair et al., 2018)	CtOs	The biallelic mutations in TSC1/TSC2 (encoding mTORC1 inhibitors hamartin/tuberin) leads to mTORC1 hyperactivation and cortical tuber formation	Pharmacological mTORC1 inhibition with rapamycin markedly reduced tuber formation
		CtOs	The newly identification of caudal late interneuron progenitor cells reveals the extended generation of interneurons in the human brain as a susceptibility for TSC	Inhibition of epidermal growth factor receptor reduces tumor burden, revealing prospective therapeutic methods for TSC and related disorders
	Focal cortical dysplasia (Xu et al., 2025)	CtOs	The existence of cardiomyocyte-like cells in cortical organoids indicates their possible heterotopic origin and transition into aberrant neurons, which may contribute to circuit hyperexcitability in focal cortical dysplasia	Cardiomyocyte heterotopia and the dissemination of neuronal hyperactivity are innovative treatment targets for focal cortical dysplasia
	Cortical development (Qian et al., 2020)	CtOs	DISC1 mutations cause deficits in cortical neuron fate specification	Understanding the molecular mechanisms underlying cortical development
Neurodegenerative disease	AD (Pomeshchik, et al., 2020; Alić et al., 2021; Fertan et al., 2024)	COs	Trisomy 21 iPSCs from Down syndrome patients were used to generate cerebral organoids that exhibited extracellular Aβ deposits, hyperphosphorylated tau (pTau), and premature neuronal loss. Conditioned media revealed elevated levels of soluble Aβ and pTau aggregates prior to the formation of histological amyloid plaques	Provided early markers for AD onset and demonstrated the protective role of the BACE2 gene against AD pathology
		HOs	Increased Aβ_42_/Aβ_40_ peptide ratios, decreased synaptic protein levels in hippocampal spheroids from AD patients carrying variations in APP or PS1 genes	Platform for developing targeted therapies for AD
	PD (Jo et al., 2016; Smits et al., 2020; Jo et al., 2021)	MOs	hMOs with functional dopaminergic neurons and neuromelanin production	Model for testing neuroprotective therapies and understanding PD pathology
			α-Synuclein aggregates and Lewy body–like inclusions from GBA1 and SNCA mutations MOs	Insights into progressive Lewy body formation and potential therapeutic targets
			LRRK2-G2019S mutation in PD reduced numbers and complexity of midbrain DA neurons, increased expression of the floorplate marker FOXA2	Understanding the role of genetic mutations in PD progression and potential therapeutic targets
	ALS (Guo et al., 2024)	SCOs	Spinal cord organoids derived from C9orf72 knockdown showed upregulation of pro-inflammatory factors	Highlighting neuroinflammation in ALS pathology
	SMA (Hor et al., 2018)	SCOs	Patients derived spinal cord organoid recapitulate motor neuron degeneration, protection by CDK4/6 inhibitors, hyperactivated endoplasmic reticulum stress, defective spliceosome function	Suggests targeting cell cycle pathways for SMA therapy
	FRDA (Ryu et al., 2025)	CerOs	CerOs derived from FRDA patient iPSCs, enriched with Bergmann glia, exhibited disease-specific phenotypes. These defects were ameliorated using histone deacetylase inhibitors or CRISPR-based genetic correction	Demonstrated the ability to recapitulate FRDA pathology and test pharmacological and genetic interventions
	MJD (Brás et al., 2022)	CerOs	Patient-derived cerebellar organoids showed impaired maturation and the presence of ataxin-3-positive aggregates, consistent with MJD pathology	Reflected disease-specific alterations and potential for modeling neurodegeneration
Psychiatric disorders	SCZ (Stachowiak et al., 2017; Shin et al., 2024)	COs	Schizophrenia patients exhibit abnormal scattering of proliferating neural progenitor cells, decreased intracortical connectivity, changes in interneurons	Insights into early developmental origins of schizophrenia and FGFR1 signaling pathways
		ThOs	22q11.2 microdeletion leads to thalamic axon overgrowth via FOXP2 dysregulation, downregulation of roundabout guidance receptor 2	Insights into neuropsychiatric disorders and potential therapeutic targets related to FOXP2 and axon pathfinding
Neuroinfection and inflammation	Enterovirus-A71 infection (Chooi et al., 2024)	SCOs	Enterovirus-A71 infection leads to motor neuron neurodegeneration through ferroptosis	Potential therapeutic strategies involving ferroptosis inhibitors such as deferoxamine
	Zika virus-induced microcephaly (Qian et al., 2016)	COs	Zika virus disrupts neural progenitor cells, leading to microcephaly	Platform for testing antiviral therapies

Aβ: Amyloid-beta; Aβ_42_/Aβ_40_: amyloid-beta peptide 42/amyloid-beta peptide 40; AD: Alzheimer’s disease; ALS: amyotrophic lateral sclerosis; APP: amyloid precursor protein; ASD: autism spectrum disorder; BACE2: beta-secretase 2; BRD4: bromodomain-containing protein 4; C9orf72: chromosome 9 open reading frame 72; CACNA1C: calcium voltage-gated channel subunit alpha 1 C; CDK4/6: cyclin-dependent kinase 4/6; CDK5RAP2: cyclin-dependent kinase 5 regulatory subunit associated protein 2; CerOs: cerebellar organoids; CLIP: class II-associated invariant chain peptide; CNVs: copy number variants; Cos: cerebral organoids; CtOs: cortical organoids; DISC1: disrupted-in-schizophrenia-1; EGFR: epidermal growth factor receptor; EV-A71: Enterovirus A71; FCD: focal cortical dysplasia; FGFR1: fibroblast growth factor receptor 1; FOXA2: forkhead box A2; FOXG1: forkhead box G1; FOXP2: forkhead box P2; FRDA: Friedreich's ataxia; GABA: gamma-aminobutyric acid; GBA1: glucosylceramidase beta 1; hMOs: human midbrain organoids; HOs: hippocampal organoids; iPSCs: induced pluripotent stem cells; LRRK2-G2019S: leucine-rich repeat kinase 2 (G2019S mutation); LTCC: L-type calcium channel; mDA: midbrain dopamine; MeCP2: methyl-CpG-binding protein 2; MJD: Machado-Joseph disease; MOs: midbrain organoids; mTORC1: mammalian target of rapamycin complex 1; PD: Parkinson’s disease; PS1: Presenilin 1; pTau: phosphorylated Tau; RhoA: ras homolog family member A; RNAi: RNA interference; ROBO2: roundabout guidance receptor 2; RTT: Rett syndrome; SCOs: spinal cord organoids; SCZ: schizophrenia; SMA: spinal muscular atrophy; SNCA: synuclein alpha; ThOs: thalamic organoids; TS: Timothy syndrome; TSC: tuberous sclerosis complex; α-synuclein: alpha-synuclein.

## Advanced Formats and Assembloids

**Figure 2:**
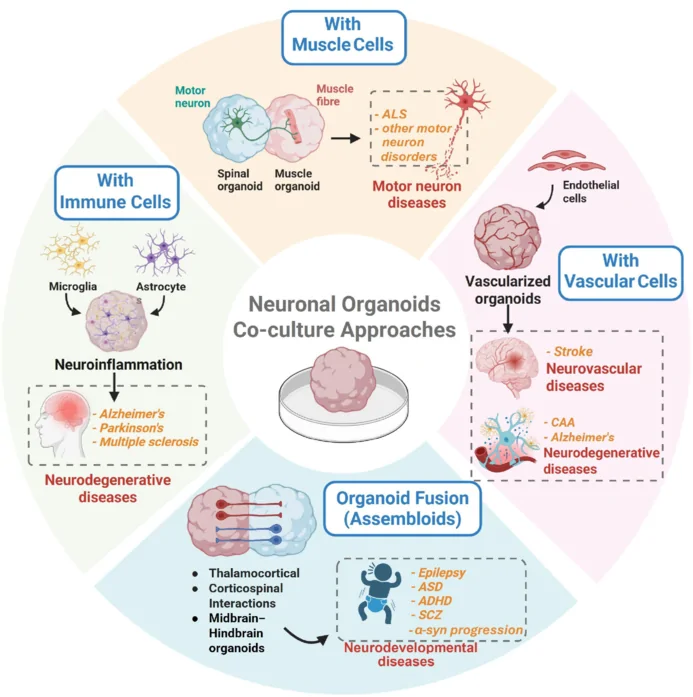
Brain organoid co-culture systems. This schematic illustrates the interactions between neuronal cells and various co-cultured cell types, highlighting key biological processes: Neuronal cells (center): Serve as the core model, used to study brain development, disease modeling, and cellular interactions in co-culture systems. Immune cells (microglia and astrocytes): Co-cultured to study neuroinflammation and cytokine signaling. Neuromuscular brain organoids: Co-culture with muscle cells enables the formation of neuromuscular junctions, facilitating studies on motor neuron connectivity and neuromuscular diseases. Vascularized brain organoids: Interaction with vascular cells (endothelial cells) supports the formation of vascular-like networks, mimicking the blood–brain barrier and improving nutrient/oxygen supply. Assembloids (thalamocortical, corticospinal, midbrain-hindbrain): Fused to study inter-regional connectivity and circuit-level dysfunction in neurodevelopmental and psychiatric disorders including epilepsy, ASD, ADHD, SCZ, and α-syn progression. Created with BioRender.com. ADHD: Attention-deficit/hyperactivity disorder; ALS: amyotrophic lateral sclerosis; ASD: autism spectrum disorder; CAA: cerebral amyloid angiopathy; SCZ: schizophrenia; α-syn: α-synuclein.

NOs have proven to be valuable resources for studying neurodevelopment and neurodegenerative diseases. However, NOs alone may not fully capture the complexity of disease phenotypes, as the involvement of non-brain cells or tissues is crucial in disease progression. Co-culturing NOs with other cells or organoids could provide a more comprehensive approach to studying these interactions. We will explore the potential of co-culturing NOs with various cells and organoids to enhance our understanding of disease mechanisms, as visualized in **Figure 2**.

### Co-culture with immune cells

The co-culture of NOs with immune cells has been explored to study neurodegenerative diseases, such as AD. Researchers have integrated microglia into NOs to investigate the role of inflammation in AD, demonstrating that immune cells can help model the neuroinflammatory environment observed in neurodegenerative conditions (Park et al., 2018). This approach allows for the examination of how immune cells contribute to disease progression, providing insights into the mechanisms driving conditions such as AD, PD, and multiple sclerosis. By including immune cells in brain organoid models, researchers can study the complex interactions between neurons and immune cells, which are critical for understanding disease pathogenesis.

The feasibility of co-culturing NOs with immune cells has been demonstrated through advances in stem cell technology and organoid culture methods. Techniques to introduce immune cells into NOs have been refined, enabling the creation of models that closely mimic the neuroinflammatory environment seen in neurodegenerative diseases. For example, protocols often involve differentiating hPSCs into NOs and then introducing microglia derived from the same or different stem cell sources (Abud et al., 2017; Ormel et al., 2018). This approach has significant potential for drug screening, allowing researchers to test potential anti-inflammatory drugs and identify those that can effectively target immune responses in the brain.

The novelty of this co-culture system lies in its ability to provide comprehensive insights into disease mechanisms and identify novel therapeutic targets. A particularly innovative aspect is the integration of patient-derived immune cells into NOs to study personalized disease mechanisms and immune responses. This could lead to the development of individualized therapeutic strategies based on a patient’s unique immune profile, offering a more targeted approach to treating neurodegenerative diseases.

### Co-culture with muscle cells

NMOs have been developed to study NMJ and motor disorders, providing valuable insights for diseases such as ALS (Gao et al., 2024). NMOs contain functional NMJs, which are essential for motor control and are often impaired in various neuromuscular diseases. These organoids can be generated from a single source of neuromesodermal progenitors derived from pluripotent stem cells. Neuromesodermal progenitors possess the intrinsic capacity to differentiate into both neural and mesodermal (muscle) lineages, enabling the concurrent formation of spinal cord neurons and skeletal muscle fibers. This approach allows for self-organization into structured organoids that recapitulate the *in vivo* integration observed during neuromuscular development (Faustino Martins et al., 2020). Alternatively, NMOs can be established using co-culture strategies, in which neural organoids and muscle cells or tissues are generated separately—often through distinct differentiation protocols—and later combined to form functional NMJs. This method employs a carefully orchestrated set of patterning cues to simultaneously direct hPSCs toward both neural and muscle fates within a shared culture system (Urzi et al., 2023). Such models are particularly useful for studying motor neuron development and function, offering a physiologically relevant platform to explore neuromuscular interactions (Faustino Martins et al., 2020; Urzi et al., 2023).

The significance of this co-culture system lies in its ability to model the neuromuscular junction in a controlled environment. Dysfunction at the neuromuscular junction is a hallmark of motor neuron diseases, and understanding the underlying mechanisms is crucial for developing targeted therapies. By co-culturing SCOs with muscle cells, researchers can investigate the formation and maintenance of neuromuscular junctions, leading to a better understanding of motor neuron diseases. This model also provides a platform for screening potential drugs and identifying those that can improve neuromuscular function.

The novelty of this co-culture system lies in its potential to reveal the underlying mechanisms of motor neuron-muscle interactions and to develop targeted therapies. A particularly innovative aspect is the potential to study the impact of genetic mutations on neuromuscular junction development and function by using patient-derived SCOs and muscle cells. This could provide insights into the specific mechanisms underlying different motor neuron diseases and lead to the development of personalized therapeutic strategies.

### Co-culture with vascular cells

Various other co-culture systems have been explored, including NOs with endothelial vascular cells to study neurovascular interactions (Cakir et al., 2019). Researchers have developed models that integrate endothelial cells into NOs to investigate the formation and function of the blood-brain barrier. The presence of these vasculature-like structures resulted in enhanced functional maturation of the organoids, including characteristics of the blood–brain barrier (BBB), such as increased expression of tight junctions, nutrient transporters, and trans-endothelial electrical resistance. This approach provided a robust model to study brain development and diseases *in vitro*, highlighting the importance of incorporating vascularization into NOs cultures to improve their viability and functionality (Shi et al., 2020; Sun et al., 2022).

Integrating vascular cells with NOs allows researchers to study various neurovascular diseases. For example, this model can be used to investigate the mechanisms underlying stroke, where the integrity of the BBB is compromised, leading to neuronal damage. Researchers can examine how vascular dysfunction contributes to the pathology of stroke and identify potential therapeutic targets to protect and repair the BBB (Cakir et al., 2019).

Additionally, the vascularized brain organoid model can be used to study diseases such as cerebral amyloid angiopathy and AD. In cerebral amyloid angiopathy, amyloid deposits accumulate in the walls of cerebral blood vessels, leading to vascular dysfunction and increased risk of hemorrhage. By incorporating endothelial cells into NOs, researchers can investigate how amyloid accumulation affects vascular integrity and function and explore potential treatments to mitigate these effects. In AD, the BBB is often disrupted, contributing to neuroinflammation and cognitive decline. The vascularized NOs model allows researchers to study these interactions in a controlled environment, providing insights into the disease mechanisms and potential therapeutic approaches.

This co-culture system also offers a platform for studying the effects of drugs on the BBB and neurovascular interactions. By using vascularized NOs, researchers can screen potential therapeutics for their ability to cross the BBB and assess their impact on both endothelial and neural cells. This can accelerate the discovery of effective treatments for neurovascular and neurodegenerative diseases (Cakir et al., 2019).

### Organoid fusion (Assembloids)

Assembloids represent an advanced iteration of co-culture approaches, combining multiple region-specific NOs to investigate inter-regional connectivity and circuitry. While traditional co-culture models focus on interactions between NOs and non-brain cells, assembloids enable the study of neural dynamics within the brain, such as thalamocortical or corticospinal interactions, providing additional layers of complexity, as depicted in **Figure 2**.

Recently, many studies have utilized assembloids to investigate NDDs by reconstructing inter-regional connectivity, interneuron migration, and neurotransmitter dynamics *in vitro*. These models recapitulate key pathophysiological features in NDDs, including excitatory/inhibitory imbalances, synaptic hyperconnectivity, and aberrant neuromodulatory signaling, providing critical insights into circuit-level dysfunctions underlying NDDs (Birey et al., 2017; Xiang et al., 2019). Specifically, recent advances have elucidated ASD-related interneuron migration deficits (Birey et al., 2022), TS-associated abnormal migratory saltations (Birey et al., 2022), SCZ-related disruptions in cortical-thalamic connectivity (Xiang et al., 2019), epileptiform hyperexcitability in patient-derived tissues (Birey et al., 2017), and attention-deficit/hyperactivity disorder-associated alterations in dopaminergic transmission (Reumann et al., 2023). By enabling precise mechanistic dissection and high-throughput drug screening, assembloids represent a powerful translational platform for personalized medicine and targeted therapeutic development in neurodevelopmental disorders (Meng et al., 2023).

One of the successful uses of assembloids has been demonstrated by Patton et al. in 2024. This innovative platform, created by merging ThOs and CtOs, reconstitutes the intricate circuitry of the thalamocortical network of the human brain. This model enables researchers to investigate synaptic plasticity, the process by which synaptic connections strengthen or weaken over time, in a human-relevant context. Given that alterations in synaptic plasticity are a key feature in the development and propagation of epileptic seizures, these assembloids provide a unique system that mimics the dynamic interactions between thalamic and cortical regions, which are critical for understanding the neural basis of epilepsy (Patton et al., 2024). The assembloid models are also used to study the cellular and circuit-level impact of neuropsychiatric disease-associated genetic variants. A recent study applied the thalamocortical assembloids to investigate the functional consequences of CACNA1G gene variants, which encode the α1G subunit of the T-type calcium channel. They found that CACNA1G mutation or loss altered T-type currents, leading to hyperactivity in thalamocortical assembloids. These results underscore the importance of T-type calcium channels in regulating thalamocortical interactions, associated with absence seizures, intellectual disability, and SCZ (Kim et al., 2024).

Another successful assembloids are cortico-motor assembloids, which are advanced 3D models created by fusing CtOs with spinal cord or muscle organoids to study connectivity between the brain and motor systems. These assembloids enable researchers to model cortico-motor interactions, such as neuronal signaling and synaptic plasticity, which are crucial for motor control. By simulating inter-regional connectivity, this platform offers unique insights into neuromuscular disorders such as ALS and motor dysfunctions (Andersen et al., 2020).

Recent work by Gomez-Giro et al. (2025) extended assembloid technology to model PD by fusing hindbrain and midbrain organoids derived from PD patient iPSCs. In line with the Braak hypothesis, which postulates two principal entry points for α-syn pathology (the olfactory bulb and the lower brainstem), followed by stereotyped propagation into midbrain nuclei, these HOs derived from patients harboring triplication of α-syn gene first developed robust α-syn protein aggregates. Upon fusion with MOs, pathogenic α-syn species progressively migrated into the midbrain compartment. This inter-regional spread was accompanied by synaptic remodeling and impaired dopaminergic neuron activity, recapitulating key functional deficits of PD. By directly visualizing both the directionality and timing of α-syn transmission, this midbrain–hindbrain assembloid model not only provides experimental support for the Braak staging framework but also offers a powerful platform for probing early-stage PD mechanisms and screening therapeutics aimed at arresting pathology before widespread neuronal loss (Gomez-Giro et al., 2025).

Assembloids, alongside simpler co-culture systems, represent a significant leap forward in our ability to model the human brain. By bridging region-specific models or integrating neural tissues with other cell types, these advanced platforms provide unprecedented opportunities to study the complex cell–cell interactions, functional connectivity, and multi-tissue pathophysiology that underlie neurological and psychiatric disorders. This approach is paving the way for a new era of targeted therapeutic development based on a more holistic understanding of brain health and disease.

## Advancing Neural Organoids for Translational Applications

Organoid approaches hold immense promises for replacing traditional therapies in patients. Here are some potential applications:

### Cell therapy and regenerative medicine

NOs have shown potential for use in regenerative medicine, including therapeutic replacement and stem cell therapy, encompassing a broad range of approaches, such as tissue engineering, aimed at repairing or replacing damaged tissues. Stem cell therapy, conversely, is a subset of regenerative medicine, specifically involves the use of stem cells to regenerate or repair tissues. NOs have been successfully used in transplantation studies in animal models, demonstrating their potential for neural repair.

The use of NOs has been demonstrated in transplantation studies using animal models. A previous study by Jgamadze et al. showed that human forebrain organoids derived from hPSCs can successfully integrate with the injured visual cortex of adult rats. The organoid grafts formed synaptic connections with the host retina and other regions of the visual system. This finding supports the potential of NO transplantation as a therapeutic strategy for restoring cortical function in cases of brain injury. The study provides evidence that human NOs can integrate both structurally and functionally with the adult mammalian brain, offering a promising approach for brain repair (Jgamadze et al., 2023).

Another study by Mansour et al. (2018) showed that NOs derived from hPSCs can be transplanted to the adult mouse brain, where organoid grafts showed progressive neuronal differentiation and maturation, gliogenesis, and integration of microglia, suggested functional synaptic connectivity between the graft and the host brain. COs have also been shown to be successfully grafted into the lesioned mouse cortex by Daviaud et al. in 2018. These grafted organoids showed robust vascularization from the host brain, facilitating nutrient and oxygen supply. The COs transplants exhibited enhanced survival compared to transplants of dissociated neural progenitor cells, demonstrating that COs transplantation represents a promising alternative to traditional cell-based transplant methods for neural repair (Daviaud et al., 2018). Zheng et al. (2023) managed to use hMOs derived from iPSCs to be transplanted into a mouse model of PD. The grafted organoids successfully integrate into the striatum’s neural circuits. Remarkably, these organoids not only restore impaired motor functions but also demonstrate potential as a functional therapy for neurodegenerative disorders. It underscores progress in using organoid models for disease treatment and neural circuit reconstruction (Zheng et al., 2023).

While NOs show promise for neural repair, other types of organoids have also demonstrated significant potential in regenerative medicine. Although there is currently no evidence of NOs being used to replace traditional therapies in patients, other types of organoids have already been utilized in regenerative medicine to generate tissues for transplantation. For example, liver organoids have been developed to study liver diseases and hold promise for use in liver transplantation (Takebe et al., 2013). Another potential application is the use of stem cells derived from organoids for stem cell therapy. Retinal organoids, for instance, have been developed to study retinal diseases and could potentially restore vision in patients with retinal degeneration by replacing damaged photoreceptor cells (Zhong et al., 2014).

### Drug discovery and screening

Organoids have emerged as a powerful tool for disease modeling and drug testing, offering a more physiologically relevant platform compared to traditional cell lines and animal models. Their ability to recapitulate the cellular composition, architecture, and functionality of human tissues makes them particularly valuable for studying complex neurodegenerative diseases such as PD. By reducing reliance on animal models, organoids not only address ethical concerns but also improve the translational potential of preclinical research (Lancaster and Knoblich, 2014). A notable example of the application of hMOs in drug testing was demonstrated by Kim et al. (2023). In their study, hMOs were utilized as a screening platform to evaluate potential therapeutic candidates targeting α-syn protein aggregation, a hallmark pathological feature of PD. Using an optogenetic system, the researchers induced α-syn aggregation in hMOs, providing a controlled and reproducible model of PD pathology. Five drug candidates, initially identified through cell line screening, were tested in this system. Among these, BAG956 and C021 dihydrochloride were found to significantly reduce α-syn aggregation in hMOs. These findings were further validated in a mouse model injected with α-syn pre-formed fibrils, where treatment with BAG956 and C021 dihydrochloride led to improved behavioral outcomes (Kim et al., 2023).

This study underscores the potential of hMOs as a robust platform for drug discovery, bridging the gap between *in vitro* and *in vivo* models. The ability to replicate key pathological features of PD and validate therapeutic candidates in both organoid and animal models highlights the translational relevance of organoid-based research. Furthermore, the use of hMOs for drug screening aligns with the growing emphasis on reducing animal testing and advancing personalized medicine, as patient-derived organoids can be used to test individualized therapeutic responses.

### Personalized medicine and target discovery

The ability to derive organoids from patient-specific iPSCs is the foundation of personalized medicine. This approach allows for the creation of *in vitro* “avatars” of a patient’s disease, enabling the study of individual disease mechanisms and the testing of tailored treatments. CRISPR-Cas9 gene-editing technology dramatically amplifies the power of this approach, moving it beyond observation to active intervention and target discovery. Gene editing allows researchers to create isogenic control lines where the disease-causing mutation is corrected in the patient’s own cells, providing a perfectly matched healthy control for experiments. Conversely, specific mutations can be introduced into healthy lines to establish causality. This is instrumental in both understanding disease mechanisms and validating therapeutic targets.

For example, Meng et al. (2023) leveraged assembloid models integrated with CRISPR-based functional genomics to systematically interrogate the impact of dozens of high-risk neurodevelopmental disorder genes. This approach allowed them to identify critical genetic networks governing neurogenesis and synaptic function, directly pinpointing novel therapeutic targets for conditions such as ASD and SCZ.

Furthermore, CRISPR-Cas9 enables genetic correction as a therapeutic strategy itself. In a landmark study, Chen et al. (2024) used ASOs, a technology closely allied with genetic manipulation, to correct aberrant splicing in Timothy syndrome-derived cortical organoids. This intervention rescued cellular phenotypes, demonstrating the potential of gene-targeting therapies. Similarly, the CHOOSE system developed by Li et al. (2023a) exemplifies how large-scale CRISPR screens in organoids can identify vulnerable cell types and fate decision pathways disrupted in disease, offering a high-throughput platform for target discovery.

By combining patient-derived organoids with precise genetic tools, researchers can now not only model diseases but also functionally validate genetic targets and test gene-based therapeutic strategies in a human-relevant context, accelerating the path towards personalized therapies for neurological disorders.

## Limitations, Challenges, and Future Directions

### Limitations and challenges

Despite significant advancements in organoid research, several critical limitations and challenges hinder the broader applicability of these models. One of the most prominent issues is batch-to-batch variability and organoid heterogeneity, both of which impact reproducibility and reliability in research and therapeutic applications.

Batch-to-batch variability arises from differences in hPSC lines, culture conditions, and slight deviations in protocols. These inconsistencies make it difficult to achieve reproducibility and standardization across studies, complicating the interpretation of results and limiting the reliability of organoid-based models (Hernández et al., 2022). This is a major obstacle for disease modeling, as it introduces significant noise that can obscure subtle but important disease-specific phenotypes, making it difficult to distinguish true pathological mechanisms from experimental artifacts.

Similarly, organoid heterogeneity in variations in cell composition and organization is driven by intrinsic differences between stem cell lines and stochastic developmental processes. This variability affects the accuracy and reliability of organoid models, underscoring the need for refined differentiation protocols and advanced characterization techniques, such as single-cell sequencing, to improve modeling fidelity (Shin et al., 2020).

Another major challenge is the long-term viability and maturation of organoids. Current protocols often fail to replicate the full complexity of adult brain architecture and function, with organoids typically resembling fetal-like tissues rather than mature brain structures (Qian et al., 2016; Bhaduri et al., 2020). Consequently, these models often fail to recapitulate key late-stage pathological hallmarks such as extensive protein aggregation (e.g., amyloid plaques, Lewy bodies) and progressive neuronal loss that define these disorders. As discussed in Section Co-Culture with Vascular Cells, the absence of functional vascular networks and the associated lack of a BBB constrain organoid size, maturation, and modeling of chronic disease processes, such as neuroinflammation, BBB breakdown in stroke or multiple sclerosis, and drug delivery across the BBB (Cakir et al., 2019; Shi et al., 2020; Sun et al., 2022). Beyond biological constraints, scalability remains a significant hurdle: producing organoids at consistent quality and scale is essential for transplantation, largescale drug testing, and achieving the statistical power needed to study rare cell types or phenotypes in discovery pipelines.

Furthermore, current organoid models have yet to successfully replicate several key forebrain regions, including the cingulate gyrus, fornix, amygdala, entorhinal cortex, claustrum, and epithalamus. The absence of these structures, which play significant roles in memory, emotion, and integration of sensory information, limits the application of organoids in studying a wide range of psychiatric (e.g., depression, anxiety) and neurological disorders (e.g., temporal lobe epilepsy, frontotemporal dementia) that critically involve these areas.

Ethical considerations also pose significant challenges. As NOs develop higher-order neuronal activity, questions about their ethical use and the necessity of defining clear boundaries for research are becoming increasingly urgent (Workman and Svendsen, 2020). From a disease modeling perspective, this ethical frontier limits how far researchers can push organoid maturation and complexity, potentially restricting our ability to fully model higher-order cognitive functions and their disintegration in psychiatric and neurodegenerative diseases. Furthermore, reproducibility concerns arise from variations in methodologies and subjective interpretations of results. These discrepancies highlight the need for standardized guidelines and collaborative efforts among researchers to ensure consistency and reliability in organoid studies.

Finally, reproducibility concerns arise from variations in methodologies and subjective interpretations of results. These discrepancies highlight the need for standardized guidelines and collaborative efforts among researchers to ensure consistency and reliability in organoid studies. A lack of reproducibility directly undermines the utility of NOs as predictive preclinical models, as findings that cannot be consistently replicated across laboratories cannot be reliably translated into therapeutic strategies for patients.

### Latest technologies in neural organoids

As NOs increasingly serve as platforms for disease modeling and drug screening, there has been significant advancement in improving both their generation and analytical methodologies. However, several challenges remain. One of the most common issues is the limited delivery of nutrients and oxygen to the center of organoids. To address this, recent articles have introduced vascular network-inspired diffusible scaffolds for midbrain organoid generation. This innovative strategy mimics the diffusion properties of natural vascular networks, resulting in enhanced dopaminergic neuron differentiation. By improving oxygen and nutrient diffusion, vascular network-inspired diffusible scaffolds help reduce necrosis, hypoxia, and cell stress, ultimately enhancing the overall function and pharmacological responsiveness of MOs (Cai et al., 2025).

Another major challenge is the complex 3D structure of NOs, which poses difficulties for accurate functional analysis. Traditional 2D electrode arrays often fall short of capturing the full spectrum of neural activity within these 3D structures. To overcome this, the development of 3D multi-electrode arrays has provided a breakthrough solution. These electrodes can wrap around, embed within, and adapt to the intricate 3D surfaces of organoids. This conformability ensures more uniform data recording across the entirety of the organoid, leading to more comprehensive and reliable functional assessments (Kim et al., 2025a, b).

Batch-to-batch variability due to differences in cell sourcing, culture conditions, and subtle environmental factors often results in inconsistent differentiation outcomes and phenotypic discrepancies between organoid batches. Addressing this, a recent study has implemented innovative multiplexing strategies that process multiple organoids simultaneously by using unique barcodes (Caporale et al., 2025).

In these approaches, each organoid is tagged with a distinct molecular barcode during sample preparation. This allows multiple organoids to be pooled for parallel processing in high-throughput analyses, such as single-cell RNA sequencing or other multiomic assays. By analyzing all samples within a single batch, researchers can minimize technical variations and normalize differences across individual runs. This not only improves reproducibility and consistency but also enhances the statistical power to detect subtle biological changes and cellular heterogeneity. Essentially, the barcode-based multiplexing strategy enables more reliable comparison across organoids by reducing the noise created by batch effects, thereby ensuring that observed differences are more likely reflective of true biological variation rather than technical artifacts (Caporale et al., 2025).

Traditional differentiation protocols for region-specific brain organoids are individually optimized through trial-and-error approaches, and the process is time-consuming and inefficient. Recent studies have demonstrated that systematically testing combinatorial temporal and dosage regimens of morphogen treatment, coupled with multiplexed single-cell transcriptomic screens by using microfluidic gradients and multi-well plate assays, can effectively identify optimal conditions for generating cell populations with defined molecular signatures. This systematic strategy significantly enhances the precision and reproducibility of region-specific NOs generation (Amin et al., 2024; Sanchís-Calleja et al., 2024).

An additional advance is the creation of a unified reference atlas for NO cell composition. He et al. (2024) combined single-cell RNA-seq datasets from multiple organoid protocols (including cortical, midbrain, and hippocampal NOs) to define conserved cell-type signatures and developmental trajectories across laboratories. By mapping cellular profiles of each organoid onto this integrated atlas, investigators can now benchmark protocol performance, detect aberrant cell populations, and guide refinement of differentiation conditions. This resource not only reduces inter-study variability by providing a common framework for cell identity annotation but also accelerates the discovery of subtle phenotypic deviations linked to disease modeling (He et al., 2024).

Overall, these advancements—from vascular network-inspired diffusible scaffolds enhancing nutrient diffusion, to 3D multi-electrode arrays improving functional readouts, to unique barcoding methods that reduce batch variability, and finally to multiplexed single-cell transcriptomic screens improving regional patterning—are pushing the field of NOs toward greater fidelity and utility in both disease modeling and drug discovery. Such innovations are critical for translating organoid-based research into robust platforms for understanding complex neurological diseases and for high-throughput therapeutic screening.

### Future directions

Despite these challenges, there are exciting opportunities for future exploration. NOs can be utilized to develop high-throughput screening platforms. This can accelerate drug discovery and toxicity testing, leading to the more efficient identification of potential therapies (Alam El Din et al., 2024), and the promising use of organoids is personalized NOs derived from patient-specific cells for personalized medicine approaches. These organoids can be used to study individual disease mechanisms and test tailored treatments (Paşca, 2016). The company Mimetas has developed the OrganoPlate platform, which facilitates 3D tissue culture under continuous perfusion in a standard 384-well plate format. This platform supports the cultivation of iPSC-derived neuronal brain tissue models, making it suitable for low- to high-throughput screening applications (Kurosawa et al., 2022). Notably, a study demonstrated the platform’s capability for high-throughput compound evaluation on 3D networks of neurons and glia, highlighting its potential in drug testing and disease modeling (Wevers et al., 2016). The brain-on-a-chip devices, which integrate microfluidics with neuronal tissues, have been developed to support high-throughput screening for drug discovery. These platforms enable precise control of the cellular environment and have been used to screen drugs, such as pitavastatin and irinotecan, in glioblastoma multiforme models (Fan et al., 2016). Additionally, these devices facilitate the modeling of the BBB, allowing for the assessment of drug efficacy in crossing this barrier (Li et al., 2023b). BBB-on-a-chip platform, a microfluidic device that replicates the BBB, has also been explored for accelerating the discovery of nanoshuttles—molecules capable of crossing the BBB for drug delivery. This technology demonstrates the ability to mimic BBB characteristics, assess molecular transport, and identify effective nanoshuttle candidates, paving the way for improved therapies for neurological diseases (Choi et al., 2024). These new techniques can be combined with NOs approaches to enhance the high-throughput screening studies.

The 3D printing techniques, particularly bioprinting, are increasingly being applied to NOs research. Bioprinting enables the precise deposition of living cells and biomaterials to create complex tissue constructs, offering enhanced control over the microenvironment and architecture of NOs. For example, the laboratory of Professor Matthias Lütolf has developed bioengineering strategies that integrate microfabrication, bioprinting, and microfluidics to improve the reproducibility, size, shape, and function of organoids. These approaches facilitate the creation of defined 3D microenvironments, which are crucial for guiding stem cell differentiation and organoid development (Brandenberg et al., 2020; Brassard et al., 2021). Additionally, advancements in tissue engineering have demonstrated the potential of 3D bioprinting to construct scaffolds and tissues with high precision. Techniques such as multi-photon processing have been employed to engineer artificial cartilage constructs, indicating the versatility of 3D bioprinting in creating various tissue types (Mačiulaitis et al., 2015; Kang et al., 2016). 3D bioprinting has also been used to construct small joint organoids for cartilage repair, offering precision in replicating the structure, mechanics, and cellular organization of joint tissues. Employing bioinks and a layer-by-layer fabrication method creates physiologically relevant models for studying joint diseases and testing therapies (Zhang et al., 2024). These developments underscore the potential of 3D printing techniques to enhance the structural and functional fidelity of NOs, thereby advancing research in neurodevelopment and neurological disease modeling.

Future research should focus on developing more sophisticated co-culture systems that better replicate the *in vivo* environment. This includes optimizing culture conditions, improving the consistency and reproducibility of organoid models, and incorporating multiple cell types. The use of microfluidic devices and organ-on-a-chip technology can provide precise control over culture conditions and simulate dynamic interactions between different cell types. Additionally, using patient-derived cells can offer personalized models for studying disease mechanisms and therapeutic responses, contributing to the development of holistic therapeutic approaches (Huh et al., 2011; Bhatia and Ingber, 2014).

A novel and promising future direction in brain organoid research is the integration of NOs with non-neural cells, such as adipocytes or hepatocytes. Research has shown cross-talk between adipocyte cells and sympathetic neurons, demonstrating that adipocyte-derived factor(s) upregulate the secretion of neuropeptides, which regulate body metabolism, food intake, and various physiological functions (Turtzo et al., 2001). Studying this interaction in 3D culture using organoids co-culture aims to provide insights into systemic interactions and their effects on brain function. By co-culturing NOs with adipocytes, researchers can explore the impact of metabolic states and obesity on brain function and neurodevelopmental disorders. This integration can provide insights into how conditions such as diabetes and metabolic syndrome contribute to neurological diseases, such as AD and depression. Co-culturing hepatocytes with glutamatergic neurons has also demonstrated that both cell types can retain their respective functionalities, such as neurotransmitter release (neurons) and metabolic enzyme activity (hepatocytes), even though their direct interactions have not been extensively investigated (Westergaard et al., 1989). This study could serve as a foundation for co-culturing NOs with hepatocytes, which may help elucidate the effects of liver health on brain function, particularly in conditions such as hepatic encephalopathy and metabolic disorders.

For NOs to serve as optimal models for age-related diseases, advancements in protocols that facilitate aging processes or preserve biological longevity are crucial. Current organoid models primarily represent early-stage neural development, lacking key hallmarks of cellular aging such as genomic instability, epigenetic drift, mitochondrial dysfunction, and protein aggregation seen in neurodegenerative diseases. To bridge this gap, future research should focus on accelerating *in vitro* aging using methods such as progeroid gene manipulation, oxidative stress induction, and long-term culturing with metabolic reprogramming to mimic late-onset neurodegenerative pathologies more effectively.

Importantly, recent work has demonstrated that COs can be used to model premature aging syndromes, such as laminopathies, and that these accelerated aging phenotypes can be pharmacologically alleviated. For example, COs derived from individuals with progeroid syndromes, including those with Down Syndrome, exhibited early aging markers, which were mechanistically characterized and therapeutically targeted, providing a proof-of-concept that age-related pathology can be reversed *in vitro* using COs (Murray et al., 2023).

Conversely, strategies to prolong organoid viability and maintain cellular homeostasis over extended periods will be essential for studying lifespan-related mechanisms in disease progression. Together, these advancements will improve the predictive power of NOs in aging research, accelerating drug discovery and personalized therapeutic strategies for age-related neurodegenerative diseases.

So far, NOs have already made significant contributions to neuroscience research. Continued advancements in this field will undoubtedly lead to a deeper understanding of the human brain and the development of innovative therapies for neurological diseases. However, as brain organoid technology advances, ethical considerations regarding their use and the extent to which they can model human consciousness and cognition will need to be addressed (Hyun et al., 2020).

## Conclusion

Different types of NOs, including cerebral, cortical, midbrain, hippocampal, thalamic, and spinal cord organoids, have been developed to model specific CNS regions and functions. NOs have revolutionized the field of neuroscience by providing a powerful tool to model human brain development, disease mechanisms, and drug testing. These 3D structures, derived from hPSC, offer significant insights into the cellular and molecular processes underlying various neurological conditions and will add to our therapeutic strategies for neurological diseases.

## Data Availability

*Not applicable.*
